# Evaluation of Orthodontic Treatment Needs Using the Dental Aesthetic Index in Iranian Students

**DOI:** 10.5812/ircmj.10536

**Published:** 2013-10-05

**Authors:** Mashallah Khanehmasjedi, Leila Bassir, Mohammad Hossein Haghighizade

**Affiliations:** 1 Department of Orthodontics, Dental School, Jundishapur University of Medical Sciences, Ahvaz, IR Iran; 2 Department of Pedodontics, Dental School, Jundishapur University of Medical Sciences, Ahvaz, IR Iran; 3 Department of Biostatistics, Health school, Jundishapur University of Medical Sciences, Ahvaz, IR Iran

**Keywords:** Malocclusion, Orthodontic Treatment, Esthetic Dental

## Abstract

**Background:**

In the contemporary orthodontics, the number of people who demand orthodontic treatment to improve their psychosocial issues related to facial esthetic is constantly increasing. Even in treatment plans, appearance and esthetic gain more attention.

**Objectives:**

The purpose of this study was to determine the need for orthodontic treatment on the basis of the dental aesthetic index (DAI) in the Iranian students of Ahvaz city.

**Material and Methods:**

This cross sectional study was performed on 900 students aged between 11 - 14 years (450 boys, 450 girls). Schools were selected based on random cluster sampling from different parts of the city. Students\ who had or were having orthodontic treatment including those on interceptive orthodontics, were excluded from the study. Two questionnaires were used; the first one included different DAI criteria, and the second one included two questions about orthodontic treatment need and satisfaction of personal dental appearance. The results were analyzed by Chi-Square and T test.

**Results:**

In 70.9% of the students, DAI score was 13 - 25, 19.2% of the samples had a DAI score of 26 - 30, the DAI score of 7.8% was 31 - 35, and in 2.1% the DAI score was greater than 35. The association between the DAI score and sex was not statistically significant (P = 0.778). In relation to orthodontic treatment need, 44.8% of students answered positively, while in 55.2% the answer was negative. In relation to satisfaction with appearance, 21% of the students were satisfied with their appearance, 59% were relatively satisfied, and 19.9% were not satisfied with their appearance. The association between DAI score and the need for orthodontic treatment and satisfaction of dental appearance was significant (P = 0.000).

**Conclusion:**

In comparison to other studies, the students in Iran (Ahvaz) have a better dental appearance and less need for orthodontic treatment. Significantly positive correlations were found between the DAI and satisfaction of dental appearance, orthodontic treatment need and student’s perception.

## 1. Background

Irregular, crowded, and protruding teeth have been a problem for some individuals since antiquity, and attempts to correct these defects date back to at least 1000 BC (1). In the 20th century, the role of dental and facial appearance was emphasized to the point that, during the last three decades, a notable increase in orthodontic treatment demand has occurred as a consequence of the high perception rate of malocclusions, along with a greater attention to esthetics. This reflects a greater awareness of the issue by parents and patients, resulting in a tendency for treatment, mostly because of their concerns on facial appearance and the related psychosocial problems. Even in treatment planning, more attention has been given to appearance and esthetics (2). Several indices have been used to indicate the need and demand for orthodontic treatment. The Dental Aesthetic Index (DAI) is one of these indices introduced for oral health research and epidemiologic studies by the World Health Organization (WHO). It is a simple, internationally accepted index, used in epidemiologic studies to evaluate the individual need for treatment. It is also considered a tool for screening treatment priority in dental public health programs (3, 4). The index expresses the esthetic and physical components of malocclusion by a single score; whereas, the other indices require separate evaluations for the aesthetic and physical components of malocclusion. In comparison with other indices, DAI is a simple and less time consuming index, which links the physical and aesthetic components mathematically to arrive at a single score (4). The DAI consists of 10 occlusal traits, each being measured for each person, and each of them is multiplied by its regression coefficient. The results are added and summed with a constant, to give the DAI score. To determine the treatment need based on the DAI score, people are categorized into four groups (5, 6). A DAI score value of 36 is used to differentiate handicapping from nonhandicapping malocclusion (5). The DAI table, weights and components are shown in Table 1. 

**Table 1.** Standard DAI Regression Equation

	DAI Components	Rounded Weight
**1**	Number of missing visible teeth (incisors, Canines and premolars teeth in the maxillary and mandibular arches)	6
**2**	Crowding in the incisal segments: (0 = no segment crowded, 1 = one segments crowded, 2 = two segment crowded)	1
**3**	Spacing in the incisal segments: (0 = no spacing, 1 = one segment spaced, 2 = two segment spaced)	1
**4**	Midline diastema, in millimeters	3
**5**	Largest anterior maxillary irregularity, in millimeters	1
**6**	Largest anterior mandibular irregularity, in millimeters	1
**7**	Anterior maxillary overjet, in millimeters	2
**8**	Anterior mandibular overjet, in millimeters	4
**9**	Vertical anterior open bite, in millimeters	4
**10**	Anteroposterior molar relationship, largest deviation from normal either left or right: (0 = normal, 1 = 1/2 cusp either mesial or distal, 2 = one full cusp or more either mesial or distal)	3
**11**	Constant	13
	Total	DAI Score

## 2. Objectives

The purpose of this study was to evaluate the orthodontic treatment need in 11 – 14 years old students in Ahvaz, Iran, for orthodontic screening and prevention programs, and to provide data that can be compared with the findings from other countries.

## 3. Materials and Methods

### 3.1. Design and Study Population

This cross sectional study was performed on 900 students aged between 11 – 14 years (450 girls, 450 boys). An official permission was obtained from the officer of the deputy director of Ahvaz public instruction office. Schools were selected based on a random cluster sampling from four districts education of the city of Ahvaz, Khuzestan province, south west of Iran. An informed consent was obtained from each pupil’s parents. The sample size calculation was performed by considering the 30% prevalence rate of orthodontic treatment need (7), with a confidence coefficient of 1.96, and a power of 80. All the 11-14 years old children of these secondary schools were examined with a single examiner until the sample size reached 900, between September and December 2008. Students, who had or who were having orthodontic treatment, including those on interceptive orthodontics, and who disagreed to participate in the study (8) were excluded from the study. For elimination of age factor the same number of each age group were selected. The study was approved by the ethnical committee of the Jundishapur University of Medical Sciences of Ahvaz, Iran (Ref. No. IA/P/8120114156 Date: 4/02/2013). A survey proforma was prepared with the help of the WHO oral health assessment form (9). To ensure the validity of the questionnaire five colleagues were asked to comment about it. Their comments and suggestions were incorporated into the final questionnaire. For reliability of the questionnaire 20 students were re-examined after two weeks, the results were tested using test-retest and the coefficient correlation was obtained 78%.

### 3.2. Examination Protocol 

The students were examined at the schools, in a quiet classroom without external interference, and need for privacy and confidentiality were stressed. Each subject was interviewed privately before being examined. The examination and interview of a single study subject took approximately 15 minutes under good natural daylight, following the WHO guidelines. The assessment of dental occlusion was performed by a single examiner, using latex gloves, dental mouth mirrors, and a Williams probe (made in the USA). A sufficient number of autoclaved instruments were carried to the examination site to avoid the interruption during the study. After each day of examination, all instruments were autoclaved. The students requiring immediate treatment were referred to the Orthodontic Department of Ahvaz University. Demographic data and information about 10 components of the DAI were obtained directly from the students and recorded in the questionnaires. The rounded weights of each component were multiplied by its corresponding regression coefficient. To reach the DAI score, the results were added and summed with a regression constant. Results were classified on the four grade scale proposed by Jenny and Cons (1996) (10), and individuals placed in levels three and four were considered to require treatment. Information on awareness of malocclusion and satisfaction with personal dental appearance was obtained from two questions on the questionnaire.

### 3.3. Statistical Methods

Student's T-test was used to compare the quantitative variables. The chi-square test was used to compare the association between the qualitative and descriptive variables. The probability value of 0.05 or less (P ≤ 0.0001) was set as the significance level. The data were analyzed using the Statistical Package for Social Science software (SPSS), version 16.0 (SPSS Inc., Chicago, Ill., USA).

## 4. Results

A total of 900 students (mean age 12.5 ± 1.118 years), comprising of 450 boys and 450 girls, of all the selected secondary schools in Ahvaz, were examined. As they classified according to the DAI index based on various malocclusion, 70.8% students had < 25 DAI scores with no or minor malocclusion requiring no or slight treatment need, 19.2% had 26 - 30 DAI scores with definitive malocclusion requiring elective treatment, 7.8% had 31 - 35 DAI scores with severe malocclusion requiring highly desirable treatment, and 2.2% had > 36 DAI scores with very severe or handicapping malocclusion requiring mandatory treatment (Table 2 and Table 3).The difference between the mean of DAI for the two genders was not statistically significant (P = 0.778) (Table 2). 

**Table 2.** Relationship Between DAI Score and Gender ^a ^

DAI, Score					
Gender	13-25, No. (%)	26-30, No. (%)	31-35, No. (%)	≥ 36, No. (%)	Total, No. (%)
**Male**	317 (70.4)	90 (20.0)	35 (7.8)	8 (1.8)	450 (100)
**Female**	32 (71.0)	83 (18.5)	35 (7.8)	12 (2.7)	450 (100)
**Total**	637 (70.8)	173 (19.2)	70 (7.8)	20 (2.2)	900 (100)

^a^ P = 0.778

**Table 3.** Relationship Between DAI and Treatment Need ^a^

DAI, Score					
Treatment Need	13 - 25, No. (%)	26 - 30, No. (%)	31 - 35, No. (%)	≥ 36, No. (%)	Total, No. (%)
**Yes**	208 (32.7)	122 (70.5)	54 (77.1)	19 (95)	403 (44.8)
**No**	429 (67.3)	51 (29.5)	16 (22.9)	1 (5)	497 (55.2)
**Total**	637 (100)	173 (100)	70 (100)	20 (100)	900 (100)

^a^ P = 0.000

In relation to the need for orthodontic treatment, 44.8% of students had answered positive and 55.2% negative (Table 2). There was a significant correlation between the DAI and treatment need (P < 0.001). 

In relation to satisfaction with the appearance, 21% of the students were satisfied with their appearance, 59.4% were relatively satisfied, and 19.6% were unsatisfied with their appearance (Table 4). 

**Table 4.** Relationship Between DAI and Satisfaction of Appearance ^a ^

DAI, Score					
Satisfaction	13 - 25, No. (%)	26 - 30, No. (%)	31 - 35, No. (%)	≥ 36, No. (%)	Total, No. (%)
**Agree**	156 (24.5)	28 (16.3)	5 (7.1)	0 (0)	189 (21)
**Relatively**	395 (62)	103 (59.4)	30 (42.9)	7 (35)	535 (59.4)
**Not Agree**	86 (13.5)	42 (24.3)	35 (50)	13 (65)	176 (19.6)
**Total**	637 (100)	173 (100)	70 (100)	20 (100)	900 (100)

^a^ P = 0.000

## 5. Discussion

The orthodontic features of different ethnic groups have been specified in several studies with the purpose of recording the prevalence of malocclusions and evaluating the orthodontic treatment need. In the present study, the DAI was used to record the orthodontic treatment need for the population and the student's perception in Ahvaz, Iran. The DAI try to combines the physical and esthetic aspects of occlusion by adding the clinical components and esthetic mathematically to produce a single score, unlike the index of orthodontic treatment need (IOTN) (11). Although the DAI has several limitations (it does not identify cases with cross bite, posterior open bite, midline discrepancy and deep overbite), its simplicity, reliability and reproducibility encourage its use as an orthodontic index (12). The potential patients’ perceptions regarding orthodontic treatment are important because the patients are the ones who receive treatment and need to gain satisfaction from improved esthetics and function (8).

In our study, 70.9% of the whole sample had DAI scores < 25, while the same score was 77.4% in Nigeria (13), 58.6% in Spain (14), 47.7% in South Africa (15), 66.5% in Turkey (16), and 3% in India (17). A total of 19.2% of students had DAI scores between 26-30; whereas, the same score was present in 21.2% of subjects in Spain (14), 20.3% in South Africa (15) that is relatively greater than Iran (Ahvaz), 12% in Turkey (16), and 15% in India (17) relatively lower than Iran (Ahvaz). A total of 7.8% had DAI scores of 31-35, the same score being encountered in 14.1% of subjects in South Africa (15), 11.2% in Spain, and 5.5% in Nigeria (13), 9.6% in Turkey (16), and 27% in India (17). Only 2.2% of our subjects had DAI scores > 36; whereas, such scores represented 9.9% in Spain (14), 16.8% in South Africa (15), 3.7% in Nigeria (13), 11.9% in Turkey (16), and 55% in India (17). The results showed that malocclusion and treatment need is greater in many countries such as Spain (14), South Africa (15), Turkey (16) and India (17) compared to Iran (Ahvaz).

In the present study, the difference between the DAI values of boys and girls indicated that boys showed a greater need for treatment than girls, but not statistically significant (Table 1). The situation is the same in Spain ( 14 ) and Nigeria ( 13 ); whereas, in Malaysia ( 18 ) the DAI score was greater in girls than boys, and the association between DAI and gender was positive and statistically significant. This study revealed significant association between orthodontic treatment need and patient subjective appreciation of treatment need (P = 0.000), which is comparable to Malaysia ( 18 ) and Brazil ( 19 ), while this association was relatively weak in Nigeria ( 13 ). Also, there was a statistically significant association between DAI and satisfaction of dento-facial appearance (P < 0.001). This association was similar to that revealed by a study performed in the USA ( 20 ), but failed to maintain its statistical strength in a study performed in Nigeria ( 13 ). 

In comparison to other studies, the students in Iran (Ahvaz) have a better dental appearance and less need for orthodontic treatment. Significant positive associations were found between the DAI and satisfaction of dental appearance, orthodontic treatment need and students’ perception. The DAI is a relatively simple, reproducible and valid index. It can be used as a practical tool for epidemiologists and other dental personnel for the screening of orthodontic treatment need.
